# Supplementary material for: Genome analysis in Avena sativa reveals hidden breeding barriers and opportunities for oat improvement
Source: Commun Biol. 2022 May 18;5:474. doi: 10.1038/s42003-022-03256-5 (PMC9117302; doi:10.1038/s42003-022-03256-5)
Supplement: Supplementary file 2 — Supplementary Information [file 42003_2022_3256_MOESM2_ESM.pdf]

Supplementary Information for:

Genome analysis in *Avena sativa* reveals hidden breeding barriers and opportunities for oat improvement

Nicholas A. Tinker[1]*, Charlene P. Wight[1], Wubishet A. Bekele[1], Weikai Yan[1], Eric N. Jellen[2], Nikos Tsardakas Renhuldt[3], Nick Sirijovski[3,4,5], Thomas Lux[6], Manuel Spannagl[6], Martin Mascher[7,8]

[1]Agriculture and Agri-Food Canada, Ottawa Research and Development Centre, 960 Carling Avenue, K.W. Neatby Bldg., Central Experimental Farm, Ottawa, Ontario, K1A 0C6, Canada.

[2]Department of Plant and Wildlife Sciences, Brigham Young University, College of Life Sciences, Provo, 84602, Utah, USA.

[3]Lund University, Department of Chemistry, Division of Pure and Applied Biochemistry, Box 124, 221 00 Lund, Sweden.

[4]CropTailor AB, c/o Lund University, Department of Chemistry, Division of Pure and Applied Biochemistry, Box 124, 221 00 Lund, Sweden.

[5]Present address: Oatly AB, Food Science, Scheelevägen 19, 223 63, Lund, Sweden.

[6]Helmholtz Center Munich – Research Center for Environmental Health, Plant Genome and Systems Biology (PGSB), Ingolstaedter Landstr. 1, 85764 Neuherberg, Germany.

[7]Leibniz Institute of Plant Genetics and Crop Plant Research (IPK), Domestication Genomics, Corrensstrasse 3, 06466 Seeland, Germany.

[8]German Centre for Integrative Biodiversity Research (iDiv) Halle-Jena-Leipzig, Puschstrasse 4, Germany.

*Correspondence

Supplementary data are contained in separate Excel files.

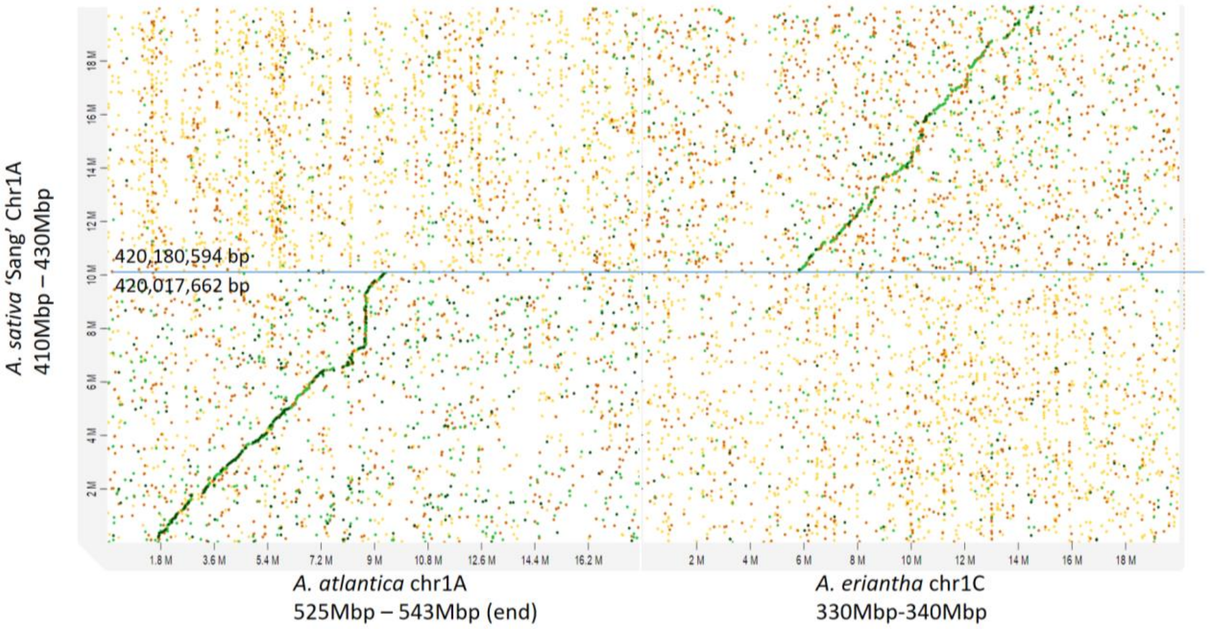

Supplementary Figure 1. Location of the large 1C→1A translocation breakpoint in the Sang reference genome. Dot plots were generated comparing the Sang genome to those of *A. atlantica* and *A. eriantha* using MiniMap and Dgenies to identify the breakpoint locations in the Sang genome.

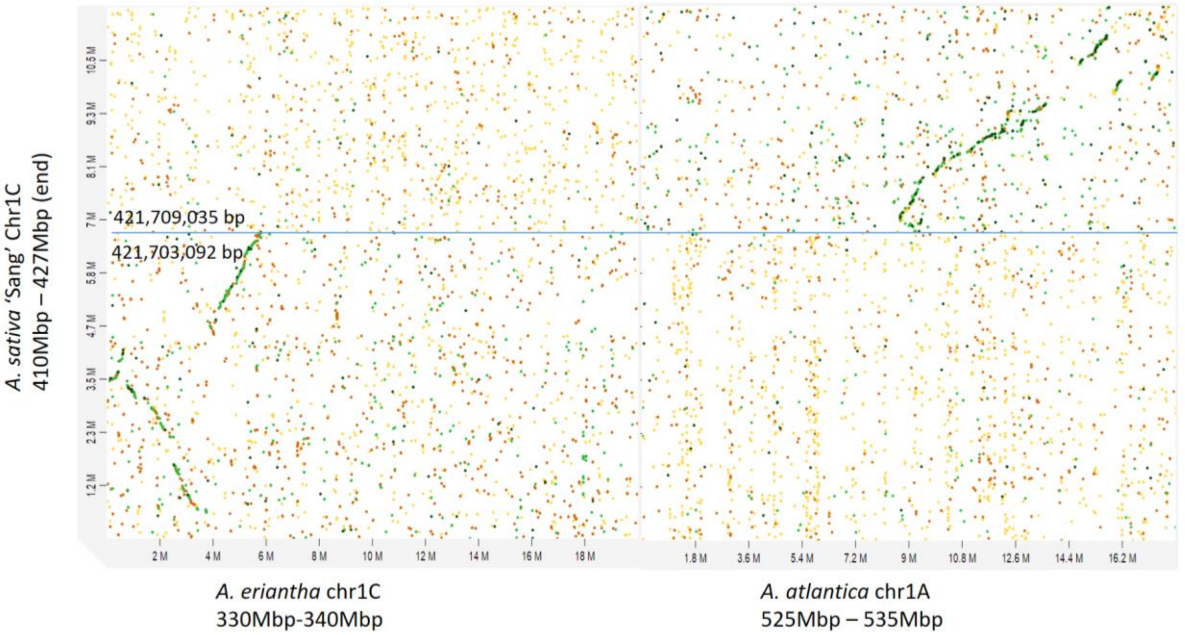

Supplementary Figure 2. Location of the small reciprocal 1A→1C translocation breakpoint in the Sang reference genome. Dot plots were generated comparing the Sang genome to those of *A. atlantica* and *A. eriantha* using MiniMap and Dgenies to identify the breakpoint locations in the Sang genome.

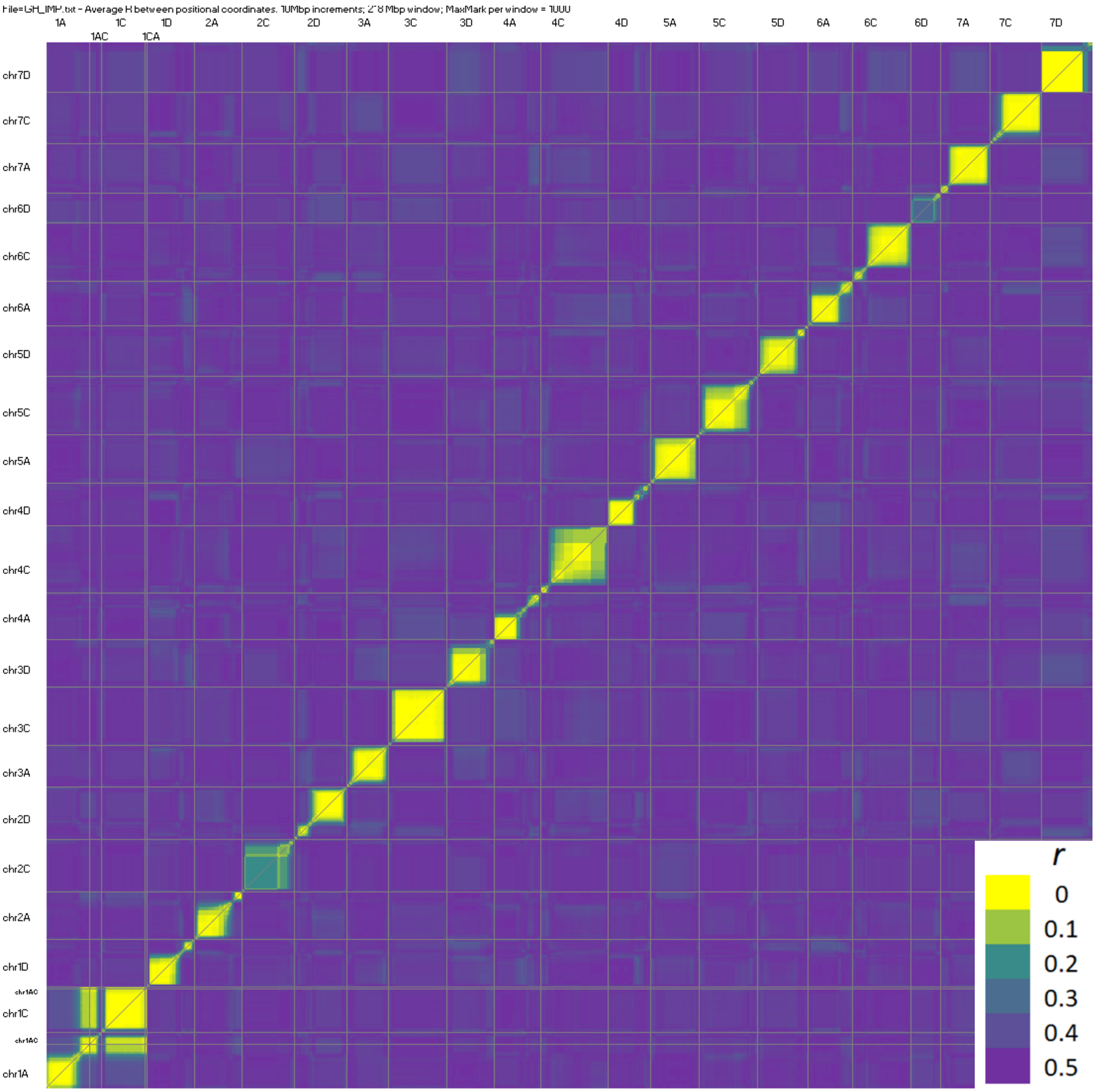

Supplementary Figure 3: Heatmap for full genome recombination matrix in population GoHF showing average pairwise recombination rates among all chromosomal regions. These were computed in 16 Mbp windows at 10 Mbp increments. The average recombination rates (r) between pairs of markers are visualized as blended colors of yellow (r=0) to teal (r=0.2) to burgundy (r=0.5). Blocks of yellow indicate recombination suppression (within a chromosome) or pseudo-linkage (between chromosomes). Pairs of positions where recombination is observed are identified by burgundy.

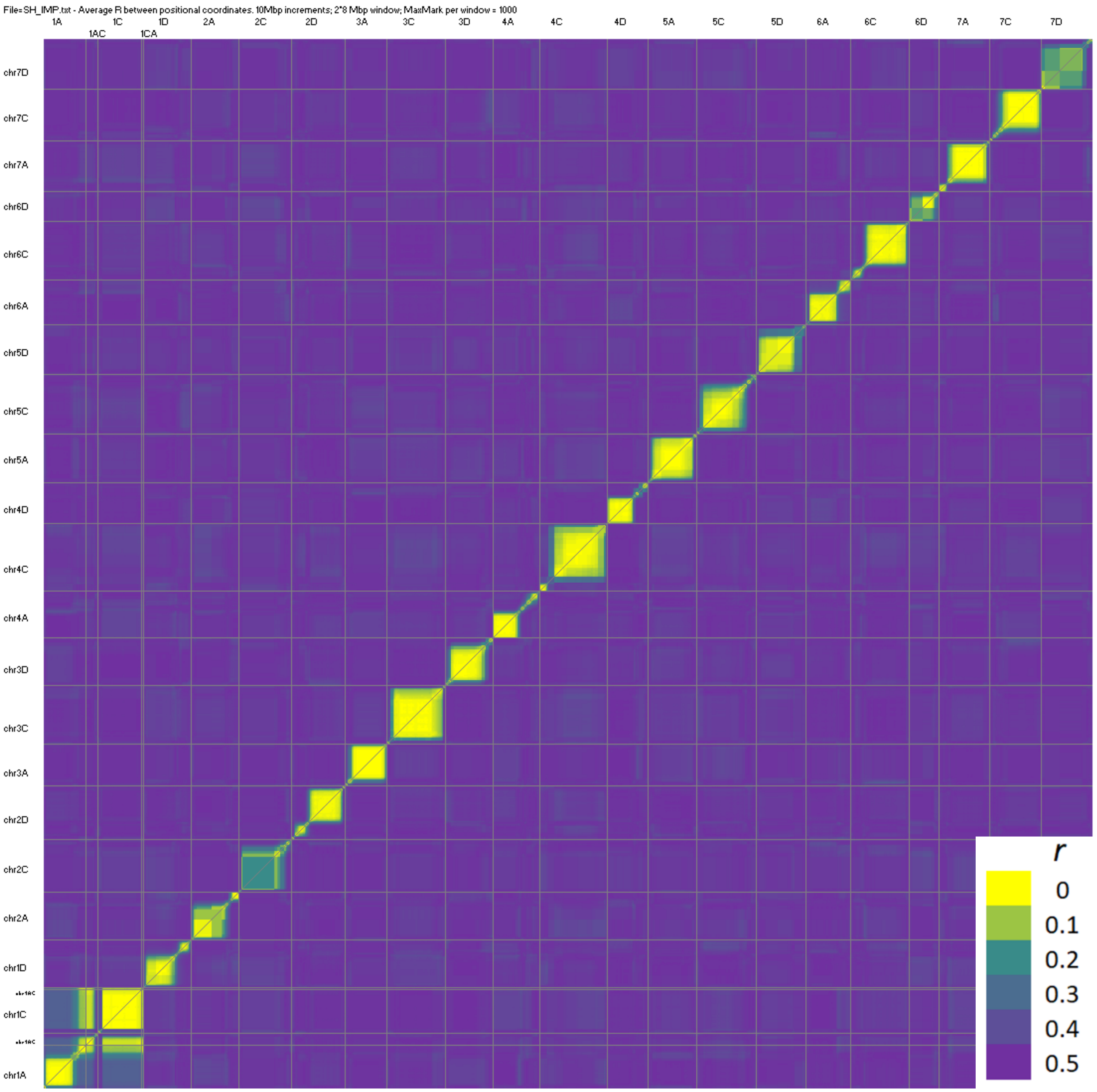

Supplementary Figure 4: Heatmap for full genome recombination matrix in population ShHF showing average pairwise recombination rates among all chromosomal regions. These were computed in 16 Mbp windows at 10 Mbp increments. The average recombination rates (r) between pairs of markers are visualized as blended colors of yellow (r=0) to teal (r=0.2) to burgundy (r=0.5). Blocks of yellow indicate recombination suppression (within a chromosome) or pseudo-linkage (between chromosomes). Pairs of positions where recombination is observed are identified by burgundy.

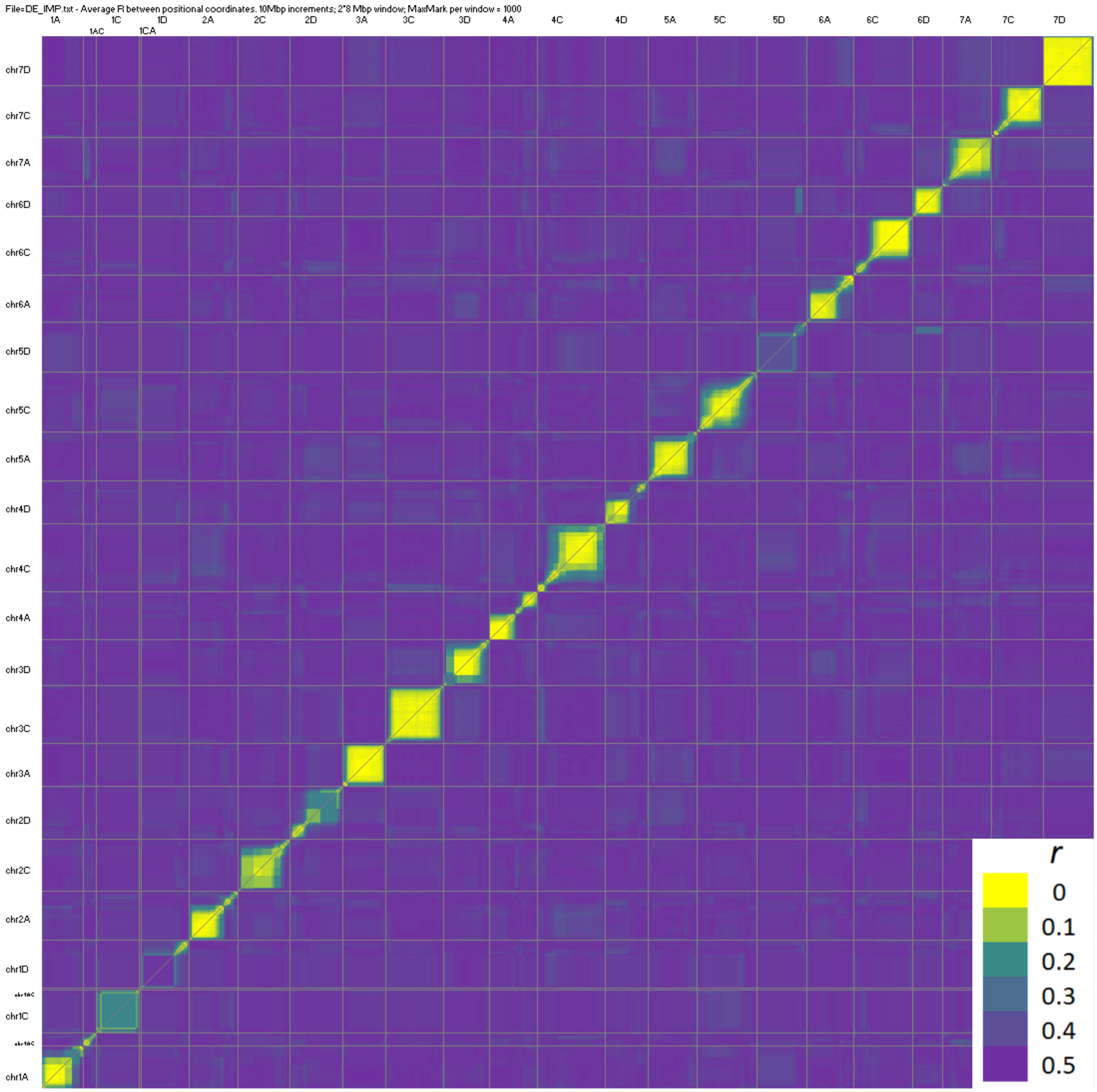

Supplementary Figure 5: Heatmap for full genome recombination matrix in population DaEx showing average pairwise recombination rates among all chromosomal regions. These were computed in 16 Mbp windows at 10 Mbp increments. The average recombination rates (r) between pairs of markers are visualized as blended colors of yellow (r=0) to teal (r=0.2) to burgundy (r=0.5). Blocks of yellow indicate recombination suppression (within a chromosome) or pseudo-linkage (between chromosomes). Pairs of positions where recombination is observed are identified by burgundy.

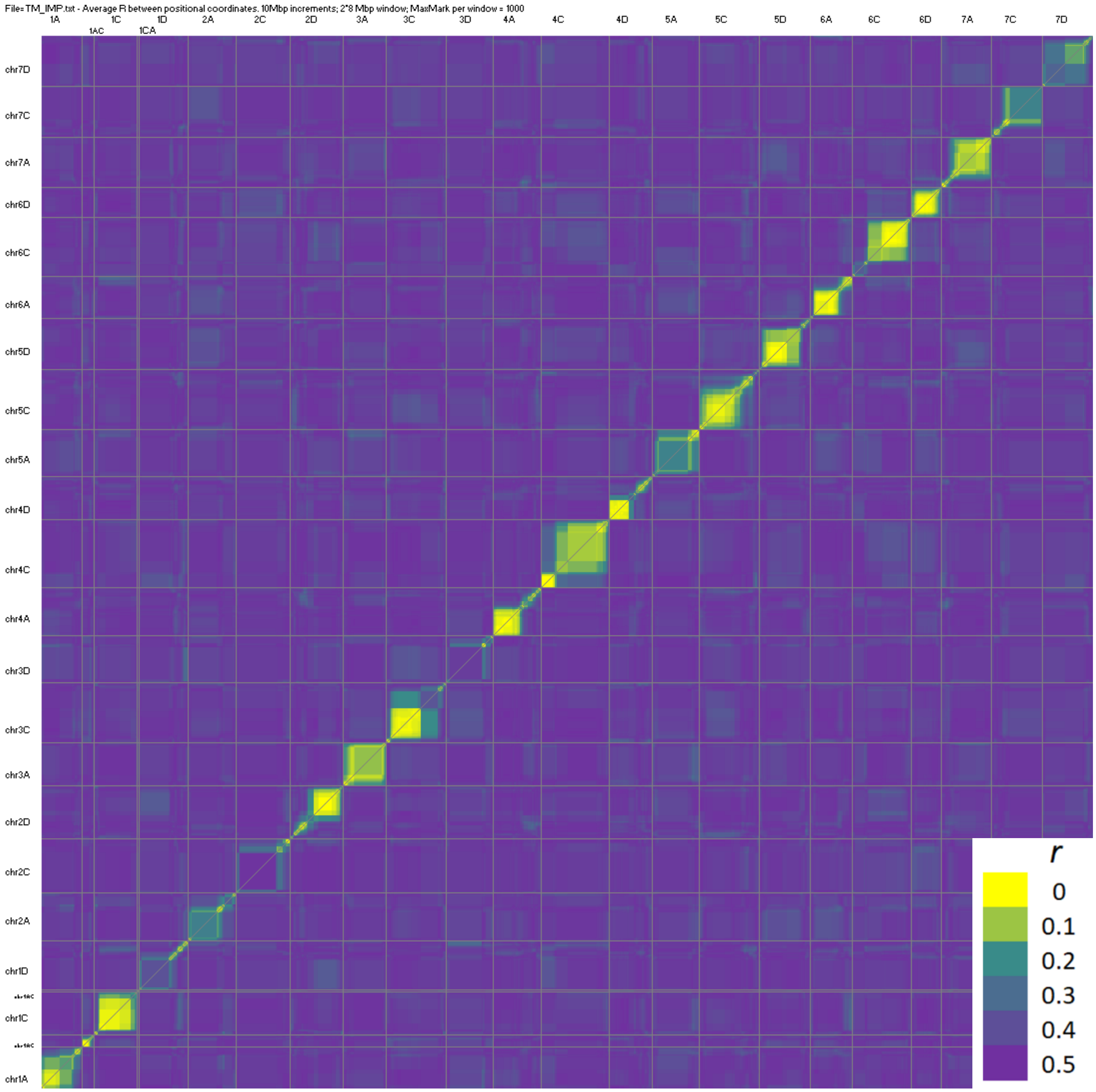

Supplementary Figure 6: Heatmap for full genome recombination matrix in population TeMa showing average pairwise recombination rates among all chromosomal regions. These were computed in 16 Mbp windows at 10 Mbp increments. The average recombination rates (r) between pairs of markers are visualized as blended colors of yellow (r=0) to teal (r=0.2) to burgundy (r=0.5). Blocks of yellow indicate recombination suppression (within a chromosome) or pseudo-linkage (between chromosomes). Pairs of positions where recombination is observed are identified by burgundy.

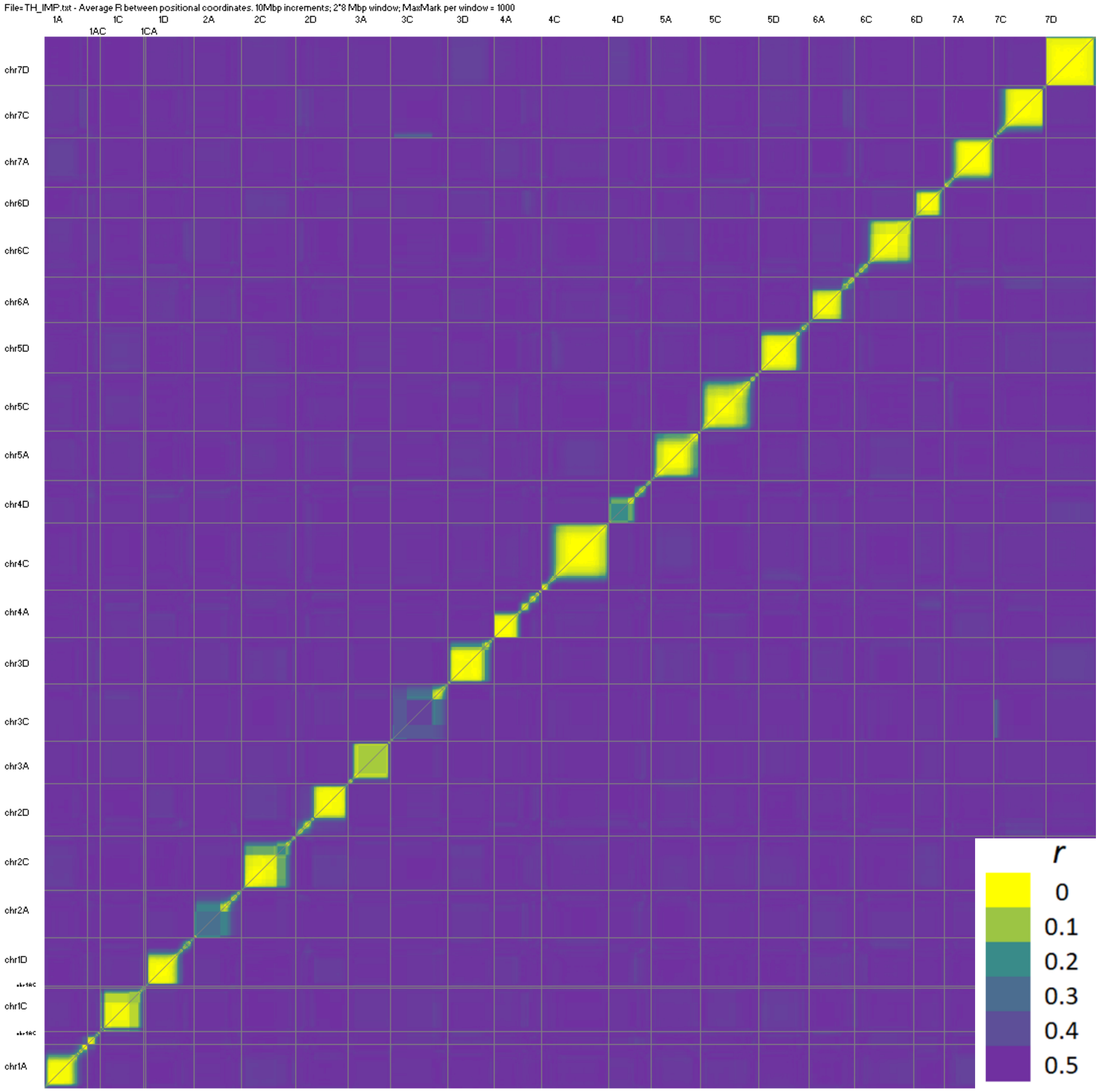

Supplementary Figure 7: Heatmap for full genome recombination matrix in population TxHd showing average pairwise recombination rates among all chromosomal regions. These were computed in 16 Mbp windows at 10 Mbp increments. The average recombination rates (r) between pairs of markers are visualized as blended colors of yellow (r=0) to teal (r=0.2) to burgundy (r=0.5). Blocks of yellow indicate recombination suppression (within a chromosome) or pseudo-linkage (between chromosomes). Pairs of positions where recombination is observed are identified by burgundy.

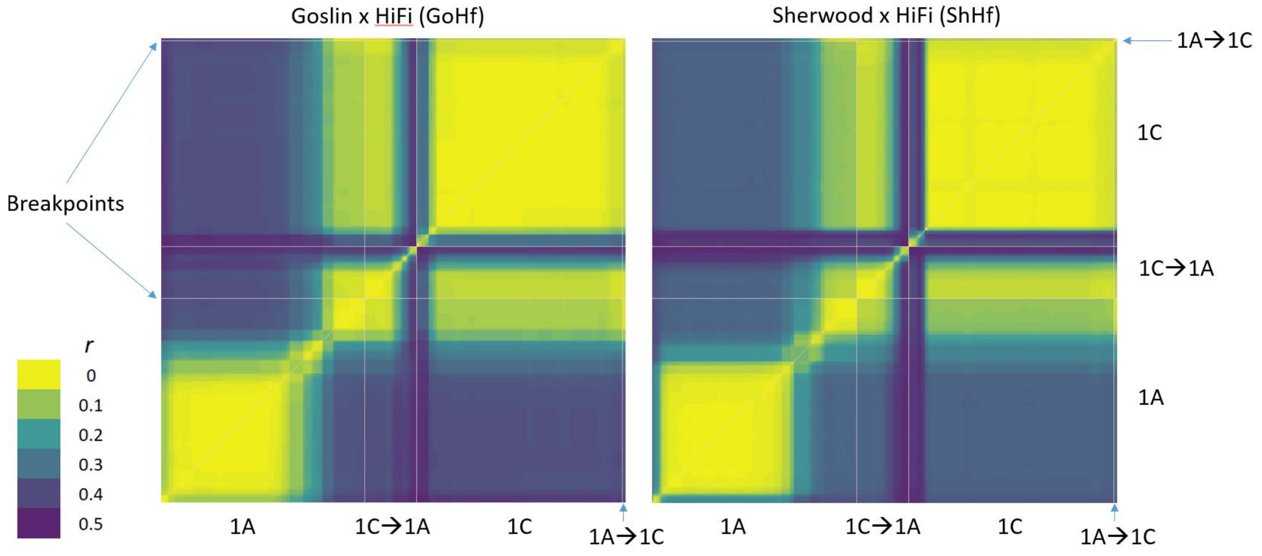

Supplementary Figure 8. Average recombination between two points in the genome based on 159 GoHf lines (left) and 207 ShHf RIL lines (right) for chromosomes 1A and 1C. These matrices are high-resolution details of those shown in Supplementary Figures 3 and 4, generated using a sliding window of 1 Mbp. These images show that GoHf and ShHf have very similar recombination profiles on these chromosomes. The image shown here for ShHf was used to generate Fig. 1 in the primary manuscript.

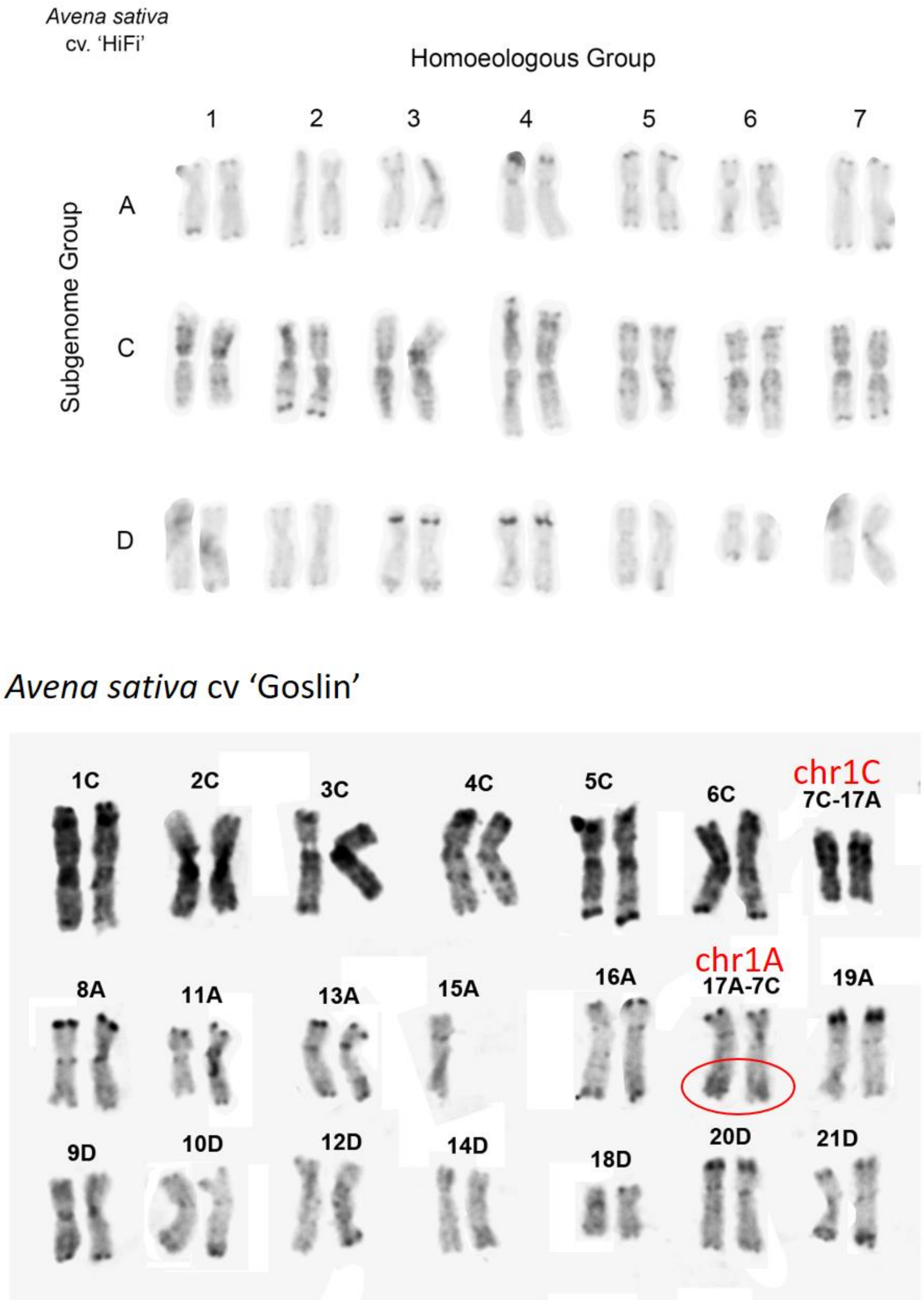

Supplementary Figure 9. C-banded karyotype of 'HiFi' Oat (top) showing the non-translocated forms of chromosomes 1A and 1C and the karyotype of 'Goslin' (bottom) showing the translocated form. Nomenclature for Goslin is based on Sanz et al. (2010)[1]; thus, the names of chromosomes 1A and 1C are updated in red. The 1C→1A translocation in Goslin is shown by a red circle. The reciprocal translocation is too small to see at the resolution available through C-banding.

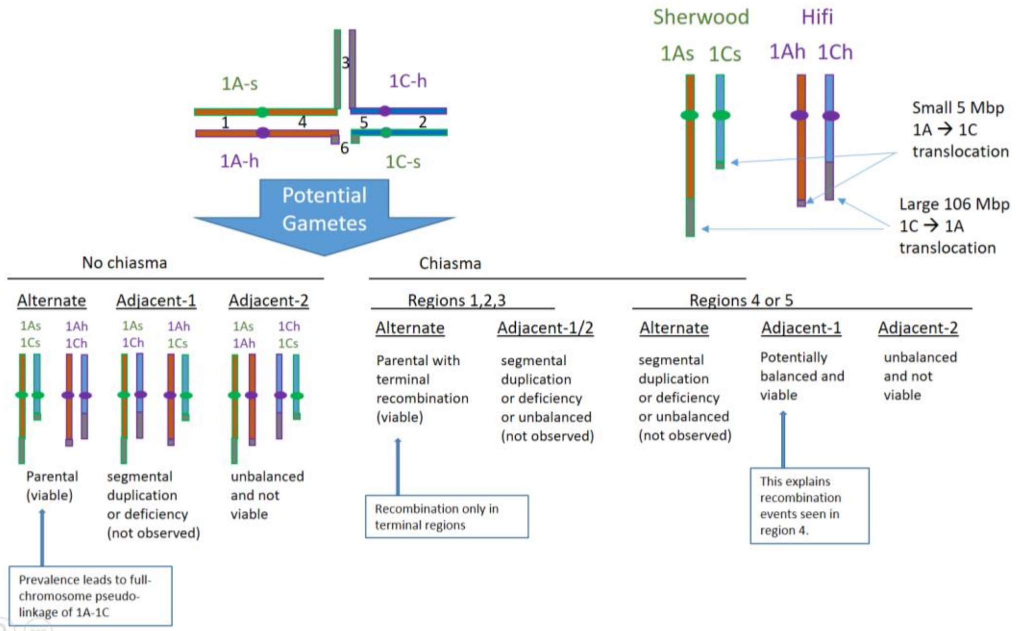

Supplementary Figure 10. Detailed model for recombination events for the unbalanced translocation between chromosome 1A and 1C in the population ShHf. This image provides additional details that supplement those shown in Figure 1 of the primary manuscript.

Supplementary References

1. Sanz M, Jellen E, Loarce Y, Irigoyen M, Ferrer E, Fominaya A. A new chromosome nomenclature system for oat (Avena sativa L. and A. byzantina C. Koch) based on FISH analysis of monosomic lines. *Theoretical and applied genetics* **121**, 1541-1552 (2010).
