# Supplementary material for: Genome analysis in Avena sativa reveals hidden breeding barriers and opportunities for oat improvement
Source: Commun Biol. 2022 May 18;5:474. doi: 10.1038/s42003-022-03256-5 (PMC9117302; doi:10.1038/s42003-022-03256-5)
Supplement: Supplementary file 3 — Description of Additional Supplementary Files [file 42003_2022_3256_MOESM3_ESM.pdf]

Description of Additional Supplementary Files

File name: Supplementary Data 1.

Description: Complete multienvironment, multi-trait phenotype data for five RIL populations.

File name: Supplementary Data 2.

Description: Raw (non-imputed) GBS marker data for five RIL populations sorted by position in the Sang reference genome.

File name: Supplementary Data 3.

Description: GBS marker data imputed by the program FSFHap in TASSEL. Details and parameters of FSFHap are provided in the legend on page 1 of the supplement.

File name: Supplementary Data 4.

Description: GBS marker data imputed by the program GBSImpute. Details and parameters of GBSi as well as Free Pascal source code are provided.

File name: Supplementary Data 5.

Description: Complete set of QTL inferences in GFF format.

File name: Supplementary Data 6.

Description: Candidate gene analysis including raw BLAST results for the Sang genome and its gene models and an interpretive summary of candidate gene positions.

File name: Supplementary Data 7.

Description: Comparative mapping of major QTLs, candidate gene loci, and historical QTLs.
