# Supplementary material for: Genome analysis in Avena sativa reveals hidden breeding barriers and opportunities for oat improvement
Source: Commun Biol. 2022 May 18;5:474. doi: 10.1038/s42003-022-03256-5 (PMC9117302; doi:10.1038/s42003-022-03256-5)
Supplement: Supplementary file 11 — Reporting Summary [file 42003_2022_3256_MOESM11_ESM.pdf]

nature research

Corresponding author(s): Nicholas A. Tinker
COMMSBIO-21-2497A_1637240994_8

Last updated by author(s): Nov 30, 2021

Reporting Summary

Nature Research wishes to improve the reproducibility of the work that we publish. This form provides structure for consistency and transparency in reporting. For further information on Nature Research policies, see our Editorial Policies and the Editorial Policy Checklist.

Statistics

For all statistical analyses, confirm that the following items are present in the figure legend, table legend, main text, or Methods section.

n/a	Confirmed	
☐	☒	The exact sample size (*n*) for each experimental group/condition, given as a discrete number and unit of measurement
☐	☒	A statement on whether measurements were taken from distinct samples or whether the same sample was measured repeatedly
☐	☒	The statistical test(s) used AND whether they are one- or two-sided<br>*Only common tests should be described solely by name; describe more complex techniques in the Methods section.*
☐	☒	A description of all covariates tested
☐	☒	A description of any assumptions or corrections, such as tests of normality and adjustment for multiple comparisons
☐	☒	A full description of the statistical parameters including central tendency (e.g. means) or other basic estimates (e.g. regression coefficient) AND variation (e.g. standard deviation) or associated estimates of uncertainty (e.g. confidence intervals)
☐	☒	For null hypothesis testing, the test statistic (e.g. *F*, *t*, *r*) with confidence intervals, effect sizes, degrees of freedom and *P* value noted<br>*Give P values as exact values whenever suitable.*
☒	☐	For Bayesian analysis, information on the choice of priors and Markov chain Monte Carlo settings
☒	☐	For hierarchical and complex designs, identification of the appropriate level for tests and full reporting of outcomes
☒	☐	Estimates of effect sizes (e.g. Cohen's *d*, Pearson's *r*), indicating how they were calculated

Our web collection on statistics for biologists contains articles on many of the points above.

Software and code

Policy information about availability of computer code

Data collection	No software was used for data collection other than standard base calling software that is part of the referenced next-generation sequencing platforms.
Data analysis	All software used for data analysis is described, provided, and/or referenced including: TASSEL-UNEAK, and Haplotag (for genotype-by-sequencing analysis), TASSEL-FSFHap and GBSimpute (for data imputation) and NQTL (for QTL analysis).

For manuscripts utilizing custom algorithms or software that are central to the research but not yet described in published literature, software must be made available to editors and reviewers. We strongly encourage code deposition in a community repository (e.g. GitHub). See the Nature Research guidelines for submitting code & software for further information.

Data

Policy information about availability of data

All manuscripts must include a data availability statement. This statement should provide the following information, where applicable:

- Accession codes, unique identifiers, or web links for publicly available datasets
- A list of figures that have associated raw data
- A description of any restrictions on data availability

There are no restrictions on data availability. All primary DNA sequence reads from the pooled GBS libraries of five RIL populations and parental lines are available in the SRA division of NCBI under BioProject PRJNA760785. Raw and imputed genotype calls and raw phenotype data are available in Supplementary Data 1 through 4. Genome sequences used for Supplementary Figs. 1 and 2 are available as referenced pseudomolecules. Data for Fig. 1 and Supplementary Figs. 3 and 4 are available in Supplementary Data 4. Data for Fig. 2 is available from Table 2, which is based on Supplementary Data 5 and 6. Results of QTL analysis in Supplementary Data 5 have been submitted to the GrainGenes19 database (https://wheat.pw.usda.gov/GG3/).

Field-specific reporting

Please select the one below that is the best fit for your research. If you are not sure, read the appropriate sections before making your selection.

- [x] Life sciences
- [ ] Behavioural & social sciences
- [ ] Ecological, evolutionary & environmental sciences

For a reference copy of the document with all sections, see nature.com/documents/nr-reporting-summary-flat.pdf

Life sciences study design

All studies must disclose on these points even when the disclosure is negative.

Sample size	Sample sizes of genetic populations were based on the available progeny which ranged from 70 to 515 for each of 5 populations. These population sizes are typical for quantitative trait locus analysis.
Data exclusions	Some progeny were dropped due to data loss or uncertainty of proper identity prior to arriving at the above population sizes. No progeny or data were dropped for selective reasons that would have biased the analyses except for the following: in year 2012, selected subsets of only 50 lines from populations GoHF and ShHF were grown. This is clearly explained in the methods, and it is dealt with by examining means for these years separately from means across remaining years where the full populations were grown.
Replication	Replication of observations were made for some field experiments, however these replications were averaged for QTL analysis. This is standard practice for QTL analysis since the effective replication is of genotypes and not progeny. e.g. in an inbred population of size 100, there are approximately 50 observations for each homozygous genotype at each locus. This concept is explained under the heading "Statistics and Reproducibility" in the Methods section.
Randomization	Progeny were randomized within each field experiment, as standard practice in field tests.
Blinding	Blinding of experimental units to observation units was performed by using randomization software for experimental design. No human intervention occurred in the randomization.

Reporting for specific materials, systems and methods

We require information from authors about some types of materials, experimental systems and methods used in many studies. Here, indicate whether each material, system or method listed is relevant to your study. If you are not sure if a list item applies to your research, read the appropriate section before selecting a response.

Materials & experimental systems

n/a	Involved in the study	
[x]	[ ]	Antibodies
[x]	[ ]	Eukaryotic cell lines
[x]	[ ]	Palaeontology and archaeology
[ ]	[x]	Animals and other organisms
[x]	[ ]	Human research participants
[x]	[ ]	Clinical data
[x]	[ ]	Dual use research of concern

Methods

n/a	Involved in the study	
[x]	[ ]	ChIP-seq
[x]	[ ]	Flow cytometry
[x]	[ ]	MRI-based neuroimaging

Animals and other organisms

Policy information about studies involving animals; ARRIVE guidelines recommended for reporting animal research

Laboratory animals	NA
Wild animals	NA
Field-collected samples	NA
Ethics oversight	NA

Note that full information on the approval of the study protocol must also be provided in the manuscript.
